# Millet Quinic Acid Relieves Colitis by Regulating Gut Microbiota and Inhibiting MyD88/NF-κB Signaling Pathway

**DOI:** 10.3390/foods14132267

**Published:** 2025-06-26

**Authors:** Sen Li, Ze Zhang, Lei Luo, Yu Zhang, Kai Huang, Xiao Guan

**Affiliations:** 1 School of Health Science and Engineering, University of Shanghai for Science and Technology, Shanghai 200093, China; lisen_1027@126.com (S.L.);; 2 National Grain Industry (Urban Grain and Oil Security) Technology Innovation Center, Shanghai 200093, China

**Keywords:** colitis, quinic acid, gut microbiota, in vitro fermentation, inflammation

## Abstract

Polyphenols are compounds derived from plant-based food possessing numerous biological activities, including inhibiting oxidative stress, suppressing inflammation, and regulating gut microbiota. In this study, we investigated the effects of quinic acid, a phenolic acid from millet, on the regulation of gut microbiota and intestinal inflammation and further discussed the possible mechanism. The results showed that quinic acid could improve the microbiota composition of the feces of patients with inflammatory bowel disease (IBD) by in vitro anaerobic fermentation by increasing the abundance of beneficial genera including *Bifidobacterium*, *Weissella*, etc., and decreasing that of harmful genera like *Escherichia-Shigella*. Quinic acid treatment could alleviate the symptoms of dextran sodium sulfate (DSS)-induced colitis in mice, maintain the intestinal barrier, down-regulate the expression of inflammatory factors such as IL-1β and TNF-α, and inhibit the activation of the MyD88/NF-κB signaling pathway. In addition, quinic acid also improved the diversity of gut microbiota in mice with colitis. Furthermore, pseudo-germ-free colitis mice proved that the effect of quinic acid on intestinal inflammation was diminished after removing most gut microbiota by antibiotic treatment, suggesting that gut microbiota play important roles during the regulation of colitis by quinic acid. In a word, our study verified the regulatory effects of quinic acid on intestinal inflammation, depending on gut microbiota regulation and NF-κB signaling suppression.

## 1. Introduction

Inflammatory bowel disease (IBD) is a chronic non-specific gastrointestinal inflammatory disease with clinical manifestations such as diarrhea, mucous purulent bloody stool, abdominal pain, weight loss, and the risk of malignant transformation [1,2]. IBD includes Crohn’s disease (CD) and ulcerative colitis (UC) [3]. Although the pathogenesis is not clear, environment, genetic factors, infection-related bacteria or viruses, abnormal activation of the autoimmune system, and complex interactions between intestinal microorganisms and hosts are considered to be related to the occurrence of IBD [4], and the high recurrence rate makes IBD become a refractory disease.

It is generally believed that IBD is related to the composition and metabolic changes in gut microbiota [5]. Compared with healthy people, the number of anti-inflammatory bacteria decreases, and the number of inflammatory bacteria increases in UC patients [6,7]. Studies on animal models further prove that the microbial community change plays a decisive role in the development of intestinal inflammation. Transferring inflammatory bacteria from diseased mice to healthy mice or colonizing gut microbiota from IBD patients into healthy germ-free mice will cause immune responses in those mice [7,8,9]. Therefore, improving the gut microbiota disorder of patients with IBD may be a potential treatment strategy.

Polyphenols are active compounds naturally generated in plants, which have anti-inflammatory, antioxidant, and probiotic effects. Thus, polyphenols may be used as adjuvants in treating IBD [10]. Cereals are a good source of dietary polyphenols. Zhang et al. [11] reported that dietary supplementation with millet suppressed the development of colitis-associated colorectal cancer and decreased the expression of IL-6 and IL-17 via inhibiting the STAT3 pathway. Another study employed fermented and germinated millet-treated colitis mice and found that the treatment could alleviate symptoms and improve gut microbiota dysbiosis [12]. Our previous study found that quinic acid is a phenolic acid component with a high content in millet, and it could improve high-fat-diet-induced neuroinflammation [13]. Studies have shown that quinic acid can arrest cell proliferation in vitro, inhibit the activation of transcription regulatory nuclear factor κB (NF-κB), and significantly increase the number of spleen white blood cells in mice to exert anti-inflammatory function [14]. Pero et al. found that quinic acid can increase the synthesis of tryptophan and nicotinamide in the gastrointestinal tract, thus enhancing DNA repair and inhibiting the activation of NF-κB by increasing the production of nicotinamide and tryptophan [15]. It has also been found that quinic acid can inhibit vascular inflammation and atherosclerosis by inhibiting the MAP kinase and NF-κB signaling pathways and the adhesion ability of vascular cell adhesion molecules [16]. Maryam et al. reported that quinic acid could ameliorate ulcerative colitis in rats and down-regulate the mRNA level of the NF-κB signaling pathways [17].

However, studies show that the intestinal absorption of quinic acid involves a strong efflux mechanism, leading to a low intestinal absorption ratio [18], while gut microbiota play an important role in its metabolism. Quinic acid is aromatized into benzoic acid by gut microbiota in vivo and then reacts with glycine in the liver and kidney to generate hippuric acid, which enters the blood [19]. Jin et al. [20] found that gut microbiota play an important role in the inhibition of atherosclerosis in ApoE−/− mice by quinic acid. In this study, we explored the potential regulatory effects of quinic acid on gut microbiota and investigated the contributions of gut microbiota to the regulation of intestinal inflammation by quinic acid. In vitro fermentation with the feces of IBD patients, a DSS-induced colitis mice model, and antibiotics-treated pseudo-germ-free colitis mice were employed, and the alteration of gut microbiota composition as well as inflammation signaling pathways were detected.

## 2. Materials and Methods

### 2.1. Materials

Quinic acid (catalogue number: B21879, purity ≥ 98%) and dextran sulfates sodium (DSS; catalogue number, v32163; molecular weight, 36~50 kDa) were purchased from Shanghai Yuanye Biotechnology Co., Ltd. (Shanghai, China). Other chemical reagents were purchased from Sinopharm Chemical Reagent Co., Ltd. (Shanghai, China). All the chemical reagents used in this study were analytically pure.

### 2.2. In Vitro Fermentation

Carbonate–phosphate buffer was prepared according to previous research, with some modifications [21]. It contained 2.00 g/L of NaHCO_3_, 3.542 g/L of Na_2_HPO_4_, 0.470 g/L of NaCl, 0.450 g/L of KCl, 0.400 g/L of urea, 0.0728 g/L of CaCl_2_·2H_2_O, 0.227 g/L of Na_2_SO_4_, 0.100 g/L of MgCl_2_·6H_2_O, 2.00 g/L of peptone, 2.00 g/L of yeast extract with 10.0 mL/L trace element containing 3680 mg/L of FeSO_4_·7H_2_O, 1159 mg/L of MnSO_4_·H_2_O, 440 mg/L of ZnSO_4_·7H_2_O, 120 mg/L of CoCl_2_·6H_2_O, 98 mg/L of CuSO_4_·5H_2_O, 17.4 mg/L of Mo_7_(NH_4_)_6_O_2_·4H_2_O, and distilled water. The pH was adjusted to 7.00. The buffer was autoclaved at 121 °C for 20 min.

Fresh stool samples from 10 IBD patients were provided by Shanghai Health Medical College (Ethical approval No. SL2021-KY-19). The inclusion criteria of the IBD patients were as follows: age of 18–70 years; diagnosed as inflammatory bowel by a clinician, currently in the remission period, and still has abdominal pain, diarrhea, and abdominal pain; and signed the informed consent form. The exclusion criteria of the IBD patients were as follows: pregnant and lactating women; used antibiotics within 4 weeks; combined with severe heart, brain, liver, hematopoietic system diseases and autoimmune diseases or other serious conditions that affect their survival; and suffered from other gastrointestinal diseases and history of gastrointestinal surgeries. The feces of the same weight from each patient were mixed and homogenized. Then, the feces were mixed with carbonic acid–phosphate buffer solution at a ratio of 1:20 (*w*/*v*) and filtered with four layers of gauze. The filtrate was collected and immediately put in an anaerobic environment (90% nitrogen, 5% carbon monoxide, and 5% hydrogen) [22]. Quinic acid was added to the mixture at a final concentration of 2 mg/mL and fermented at 37 °C in an anaerobic incubator. OD_600_ and pH were detected at 0, 6, 12, 24, and 48 h. Meanwhile, 2 mL samples were taken at 24 h for 16S rRNA analysis.

### 2.3. Animals

Male BALB/C mice (aged 6–8 weeks, weight 20 ± 2 g) were purchased from Shanghai Jiesijie Laboratory Animal Co., Ltd. (Shanghai, China). All mice were allowed free access to tap water and maintenance feed. The room was kept for 12 h in the daytime and 12 h in the dark, and the room temperature was maintained at 22–24 °C. All animal procedures were performed by the Guidelines for Care and Use of Laboratory Animals Act of the People’s Republic of China, and the experimental animal ethical approval No. was IRB-AF63-V1.0.

### 2.4. Experimental Design

DSS-induced colitis model: After 1-week adaptation, 32 mice were randomly divided into 4 groups (n = 8 per group): control group (C), DSS-induced colitis group (D), low-dose (10 mg/kg) quinic acid-treated group (QL), and high-dose (30 mg/kg) quinic acid-treated group (QH). From the first day of DSS induction, the D, QL, and QH groups were treated with 2.5% (*w*/*v*) DSS (added in drinking water), while the C group was given normal water. At the same time as the DSS treatment, the QL and QH groups were given quinic acid gavage, which was diluted in saline, and the C and D groups were given the same volume of normal saline gavage. The dose of quinic acid employed in this study was a pharmaceutical dose of quinic acid for animals and humans, higher than provided by consuming millet. The treatment lasted for 12 days, and the mice were sacrificed after 12 h fasting. Pseudo-germ-free colitis mice model: After 1-week adaptation, 32 mice were randomly divided into 4 groups (n = 8 per group): control group (C), DSS group (D), pseudo-germ-free mice group (DSS + ABX), and pseudo-germ-free quinic acid gavage group (DSS + ABX + Q). Except for the C and D groups, the other two groups of mice were free to drink water mixed with antibiotics (1.0 g/L neomycin, 1.0 g/L metronidazole, 1.0 g/L ampicillin, and 0.5 g/L vancomycin) for 7 days. After the pseudo-germ-free mouse model was successfully established, except for the control group, the other mice were treated with 2.5% DSS for 12 days. Simultaneously, the DSS + ABX + QH group was given 30 mg/kg quinic acid once a day by gavage, while the C, D, and DSS + ABX groups were given the same volume of sterilized saline. The fresh feces of mice after antibiotic treatment for seven days were collected, and the total DNA of gut microbiota was extracted according to the rapid extraction kit of bacterial genomic DNA, and the concentration was determined by a spectrophotometer. Fresh feces were also subjected to plating culture to verify the construction of the pseudo-germ-free model. A corresponding volume of sterilized saline was added according to the weight of feces (0.02 g/mL). The mixture was diluted with sterile saline by an equal volume of 10^4^–10^7^ CFU/mL and cultured on plate counting agar at 37 °C in an anaerobic incubator. The total number of colonies after anaerobic culture for 48 h was measured.

At the end of the experimental period, the mice were fasted for 12 h, and blood samples were collected from the eyeballs of the mice for subsequent analysis. The mice were sacrificed by neck-breaking, and the colonic tissues and cecum of the mice were collected, respectively, and immediately frozen with liquid nitrogen and stored in a refrigerator at −80 °C.

### 2.5. Evaluation of the Disease Activity Index (DAI)

The DAI was determined based on three parameters: body weight loss score, stool consistency, and fecal occult blood degree. The weight of the mice, the state of the stool, and the fecal occult blood degree were recorded daily. The rating scales are shown in Supplementary Table S1, and the DAI was calculated as follows [23]:

DAI = (aggregate score of weight loss, stool consistency, and bleeding)/3.

### 2.6. Histopathological Analysis of the Colon

After the mice were killed, the tissue samples from the distal colon were fixed with 4% formalin for 48 h, dehydrated with graded alcohol (75–100%) solution, and then embedded in paraffin, sectioned at 4 μm, and stained with hematoxylin and eosin (H&E). Photographs were taken by microscope, and histological scores were performed by previously reported methods [23].

### 2.7. Reverse Transcription Quantitative Polymerase Chain Reaction (RT-qPCR)

A small segment of colon tissue was isolated and homogenized. Total RNA of the colonic sample was extracted by using Magzol reagent (Vazyme Biotech Co., Ltd., Nanjing, China), and reverse transcription of the RNA was performed using a Hiscript II Q RT Supermix for qPCR (+gDNA wiper) (Vazyme Biotech Co., Ltd., Nanjing, China). Real-time PCR was performed using SYBR Green PCR Core Reagent (Vazyme Biotech Co., Ltd., Nanjing, China) in the Thermo Life Tech ABI Quant Studio system. GAPDH was used as an internal reference gene, and the relative mRNA level of TNF-a, IL-1b, and Occludin was calculated by the 2^−ΔΔCt^ method. All primer sequences are shown in Supplementary Table S2.

### 2.8. Western Blot Analysis

Proteins were extracted from colon tissue, which was homogenized in cell lysis buffer for Western and IP (Beyotime Biotechnology, Co., Ltd., Shanghai, China). After homogenization, the homogenate was centrifuged at 10,000× *g* for 10 min at 4 °C, and the supernatant was collected. Protein concentration was measured by a BCA protein kit (Beyotime Biotechnology Co., Ltd., Shanghai, China). Then, the proteins were separated by SDS-PAGE and transferred to a polyvinylidene difluoride (PVDF) membrane. After blocking with 5% skim milk for 2 h, the membrane was incubated with appropriate diluted antibody at 4 °C overnight. The specific antibodies used are as follows: anti-TNF-α (1:1000, Abcam), anti-TNF-α (1:1000, Abcam), anti-Occludin (1:1000, Affinity), anti-MyD88 ((1:1000, Affinity), anti-NF-κB and anti-pNF-κB (1:1000, Beyotime), anti-IκB-a and anti-pIκB-a (1:1000, Affinity), and anti-β-actin (1:1000, Beyotime). After that, the membrane was washed with TBST four times each time for 15 min. Then, horseradish peroxidase-conjugated goat anti-rabbit or anti-mouse IgG was added and incubated for 2 h (1:5000, Beyotime Biotechnology Co., Shanghai, China), and an ECL Substrate Kit (Beyotime Biotechnology Co., Shanghai, China) was used for detecting protein bands. The ChemiDoc Imaging system (Bio-Rad Co., Shanghai, China) was used for imaging, and Image Lab 5.0 software was used for quantitative analysis.

### 2.9. 16S rRNA Sequencing

Samples of mouse cecum feces and in vitro fermented feces from colitis patients were prepared for microbiome analysis. Fecal DNA was extracted using an E.Z.N.A. stool DNA Kit (Omega Bio-tek, Norcross, GA, USA) according to the manufacturer’s instructions. The region V3-V4 of the bacterial 16S rRNA gene was amplified with primer pairs 338F (5′-ACTCCTACGGGAGGCAGCAG-3′) and 806R (5′-GGACTACHVGGGTWTCTAAT-3′). The 16S rDNA gene was amplified, and the PCR products were extracted from 2% agarose gel, purified by an AxyPrep DNA gel extraction kit (Axygen biosciences, Union City, CA, USA), and quantified by a Quantus™ fluorometer (Promega, Madison, WI, USA). The purified PCR products were sequenced on the Illumina MiSeq PE300 platform (Illumina, Foster City, CA, USA) of Majorbio Bio-Pharm Technology Co., Ltd. (Shanghai, China).

The resulting sequences were quality filtered with fastp (0.19.6) and merged with FLASH (v1.2.11). Then, the DADA2 plugin in the Qiime2 (version 2020.2) pipeline with recommended parameters was used to obtain the de-noised high-quality sequences. Taxonomic assignment of sequences was performed using the Naive bayes consensus taxonomy classifier implemented in Qiime2 and the SILVA 16S rRNA database. UPARSE version 7.1 was used to cluster operation classification units, and the similarity was 97%. The analysis of α, Principal Co-ordinates Analysis (PCoA), Venn diagram, and linear discriminant analysis effect size analysis (LEfSe) were carried out through the online platform of https://cloud.majorbio.com/, accessed on 9 November 2022.

### 2.10. Statistical Analysis

All data are expressed as mean ± SEM. Statistical analysis was performed with GraphPad Prism 8.0.2 (GraphPad Software, San Diego, CA, USA). Differences among the experimental data were assessed by one-way ANOVA, followed by Tukey’s post hoc multiple comparison test. *p* ≤ 0.05 was considered statistically significant.

## 3. Results

### 3.1. Quinic Acid Improved the Microbial Composition of IBD Patients by In Vitro Fermentation

**Figure 1:**
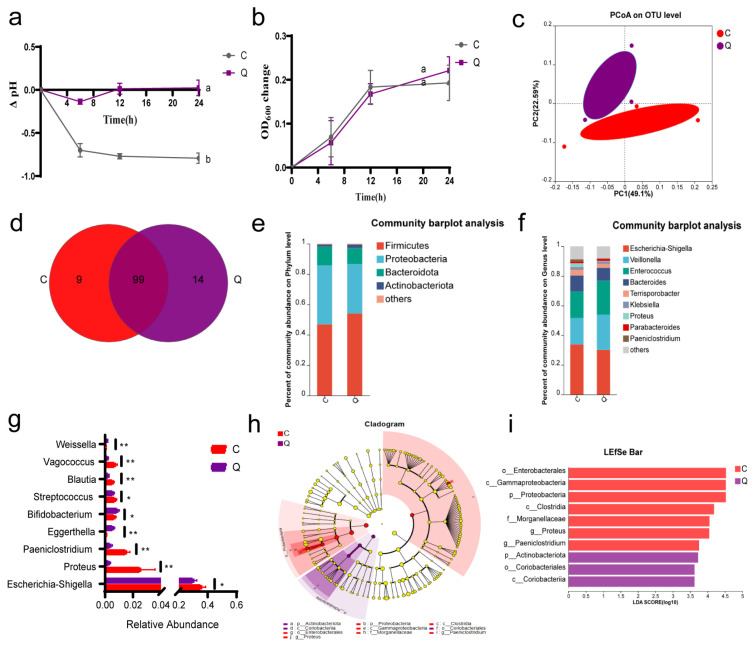
Effects of quinic acid on the gut microbiota of IBD patients by in vitro fermentation. (**a**) Change in pH—different letters (a and b) indicate significant differences at *p* < 0.05; (**b**) change in OD600; (**c**) PCoA plots; (**d**) Venn diagram of species at the OTU level; (**e**) column diagram of microbial composition at phylum level; (**f**) column diagram of microbial composition at genus level; (**g**) relative abundance of altered bacteria at genus level, * *p* < 0.05, ** *p* < 0.01; (**h**) taxonomic tree of linear discriminant analysis (LDA) effect size (LEfSe) analysis; (**i**) histogram of LDA scores higher than 3 calculated from feature differential species.

*In vitro* fermentation was utilized to investigate the regulatory effects of quinic acid on gut microbiota. The feces of IBD patients were mixed with quinic acid and fermented in an anaerobic environment. During fermentation, the alteration of the pH value in group Q showed a different trend from that in group C (Figure 1a). The pH value decreased in group C in the first 6 h and then tended to be stable, but the pH value in group Q showed no apparent change during the fermentation, which may indicate the growth of different microbes after quinic acid treatment. At the same time, the absorbance of fermentation broth at 600 nm was measured to determine the bacterial density. In the first 12 h, the OD_600_ value of the two groups increased rapidly, and after 24 h of fermentation, there was no significant difference in OD_600_ values between group C and group Q (Figure 1b).

The changes in microbiota after fermentation with quinic acid for 24 h were determined by 16S rRNA sequencing. Principal Co-ordinates Analysis (PCoA) was used to describe the diversity of different microbial communities at the OTU level (β-diversity). According to the PCoA graph, it could be observed that the microbiota of group C and group Q were obviously separated (Figure 1c), indicating there was a significant difference in the β-diversity between the two groups. The Venn diagram showed that there were nine unique genera in group C and 14 unique genera in group Q (Figure 1d).

The relative abundance of fecal bacteria was further analyzed at the phylum and genus levels. At the phylum level, the dominant species of the two groups were mainly *Firmicutes*, *Proteobacteria*, *Bacteroidota*, and *Actinobacteriota*, and quinic acid fermentation led to a decrease in *Proteobacteria* and an increase in *Firmicutes* and *Actinobacteriota* (Figure 1e). According to the histogram at the genus level (Figure 1f), the dominant floras of the two groups were *Escherichia-Shigella*, *Veillonella*, *Enterococcus*, *Bacteroides*, *Terrisporobacter*, *Klebsiella*, *Proteus*, *Parabacteroides*, etc. From Figure 1g, it could be observed that the abundance of *Escherichia-Shigella*, *Proteus*, *Paeniclostridim*, *Streptococcus*, *Blautia*, and *Vagococcus* was decreased in group Q, and that of *Eggerthella*, *Bifidobacterium*, and *Weissella* was increased compared with group C.

From linear discriminant analysis effect size (LEfSe) analysis (Figure 1h,i), group C was predominantly enriched for *o__Enterobacterales*, *c__Gammaproteobacteria*, *p__Proteobacteria*, and *c__Clostridia*, while group Q was enriched for *p__Actinobacteriota*, *o__Coriobacteriales*, and *c__Coriobacteriia***.** These results indicate that quinic acid could obviously change the diversity and abundance of fecal flora of IBD patients by fermentation.

### 3.2. Quinic Acid Alleviated the Symptoms of DSS-Induced Colitis

**Figure 2:**
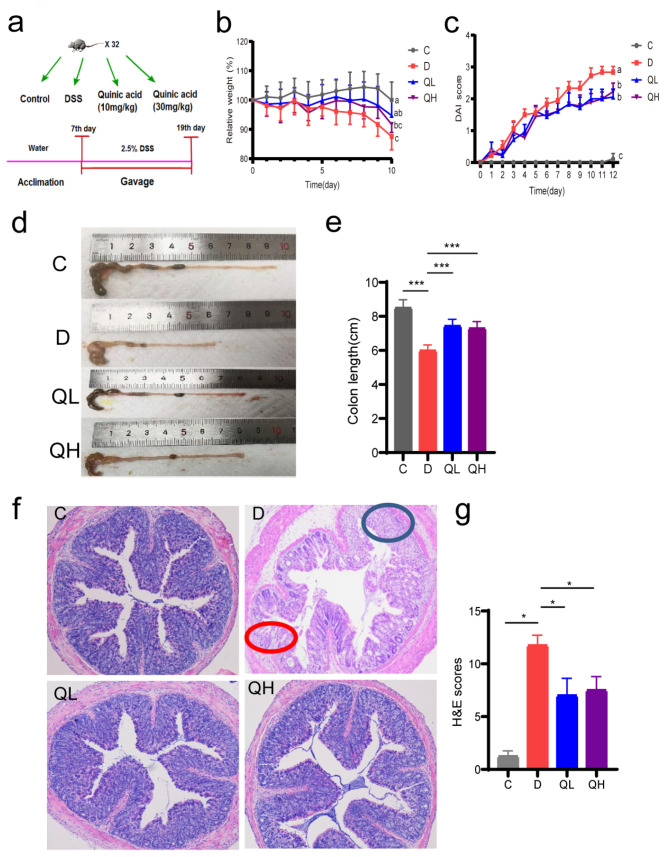
Quinic acid alleviated the symptoms of DSS-induced colitis in the mice. (**a**) Experimental scheme; (**b**) rate of weight change—a different letter of each line represents significant differences (*p* < 0.05); (**c**) disease activity index (DAI), a different letter on each line indicates significant difference at *p* < 0.05; (**d**) the pictures of colons; (**e**) colon length; (**f**) H&E pictures of colons, blue circle—inflammatory cell infiltration, red circle—destroyed crypt structure; (**g**) H&E scores. The results are expressed as mean ± SEM, * *p* < 0.05, *** *p* < 0.001.

To evaluate the regulating effect of quinic acid on the symptoms of colitis, we employed a DSS-induced colitis mice model (Figure 2a). Loss of body weight, shortened colon length, and increased DAI values are the main symptoms of colitis. The results showed that DSS induction significantly resulted in weight loss, rapidly increased DAI value, and significantly shortened colon length compared with the control group (Figure 2b–e). However, quinic acid treatment significantly alleviated the weight loss of the mice, improved the DAI scores, and significantly increased the colon length of the mice (Figure 2b–e), suggesting that quinic acid treatment significantly improved the symptoms of DSS-induced colitis in the mice.

To evaluate the effect of quinic acid on histological changes in the DSS-induced colitis mice, H&E staining was used to observe the colon tissue of the mice. As shown in Figure 2f, the morphology of the colon was normal in the control group, which showed intact colonic mucosa, regularly arranged glands, normal crypts, and no ulcer or inflammatory cell infiltration. After DSS induction, severe acute colitis occurred, with the basic structure and crypt structure destroyed. In addition, the mucus layer almost disappeared, and a large number of infiltrated inflammatory cells were found in the submucosa and muscle layer (Figure 2f). The pathological indexes of the two quinic acid-treated groups were significantly improved, with a slight inflammatory reaction, basically complete tissue structure, normal goblet cells, and no inflammatory cell infiltration (Figure 2f). Histological scores showed that quinic acid could improve the colon pathological changes induced by DSS (Figure 2g). These results indicate that quinic acid could effectively reduce the injury of colon tissue induced by DSS in the mice.

### 3.3. Quinic Acid Suppressed Intestinal Inflammation and Up-Regulated Barrier Protein Expression

**Figure 3:**
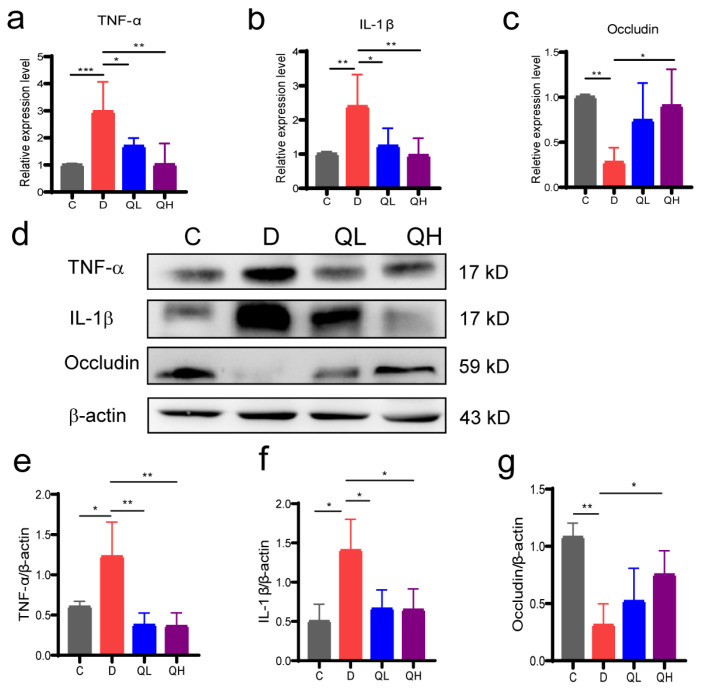
Effects of quinic acid on inflammation in colonic tissue. (**a**) Relative mRNA level of TNF-α; (**b**) relative mRNA level of IL-1β; (**c**) relative mRNA level of Occludin; (**d**) protein expression levels of TNF-α, IL-1β, and Occludin; (**e**) quantitive analysis of the protein level of TNF-α; (**f**) quantitive analysis of the protein level of IL-1β; (**g**) quantitive analysis of the protein level of Occludin. The results are expressed as mean ± SEM, * *p* < 0.05, ** *p* < 0.01, *** *p* < 0.001.

To determine the regulation of quinic acid on intestinal inflammation and barrier, the mRNA and protein levels of inflammatory factors and intestinal barrier proteins were analyzed by qPCR and Western blotting. Compared with the control group, the expression of the inflammatory factors TNF-α and IL-1β in the DSS group increased at the mRNA level. In contrast, the expression of these inflammatory factors in the quinic acid-treated groups was down-regulated compared with the DSS group (Figure 3a,b). The mRNA level of Occludin, a tight junction protein, was decreased by DSS and restored by quinic acid (Figure 3c). The protein levels of TNF-α, IL-1β, and Occludin showed similar alteration among the different groups (Figure 3d–g). These results indicate that quinic acid treatment suppressed DSS-induced intestinal inflammation and improved the intestinal barrier.

### 3.4. Quinic Acid Regulated the NF-κB Signaling Pathway in DSS-Induced Colitis Mice

**Figure 4:**
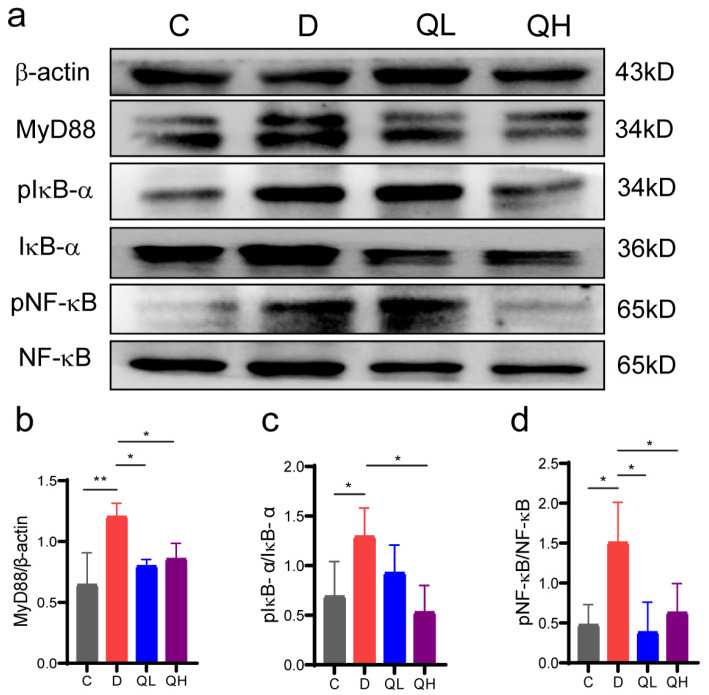
Quinic acid suppressed the MyD88/NF-κB signaling pathway. (**a**) Protein levels of MyD88, IkB-α, pIkB-α, NF-κB, and pNF-κB; (**b**) quantitive analysis of the protein level of MyD88; (**c**) quantitive analysis of the protein level of pIkB-α; (**d**) quantitive analysis of the protein level of pNF-κB. The results are expressed as mean ± SEM, * *p* < 0.05, ** *p* < 0.01.

NF-κB is a transcription factor that controls the transcription of inflammatory cytokines, such as TNF-α, IL-6, and IL-1β, and can be activated by upstream signals such as TLR4. The TLR4/MyD88/NF-κB signaling pathway is an important inflammation regulatory pathway. To investigate whether quinic acid inhibited DSS-induced colitis inflammation through the MyD88/NF-κB signaling pathway, the expression levels of key factors of this signaling pathway, such as MyD88, NF-κB/pNF-κB, and IκB-α/pIκB-α, were detected. From Figure 4, the expression level of MyD88 protein and the phosphorylation levels of NF-κB and IκB-α (pNF-κB, pIκB-α) in the colon tissue of the mice were significantly increased after DSS induction in comparison with group C, suggesting the activation of the MyD88/NF-κB signaling pathway. After quinic acid treatment, the expression level of MyD88 protein and the phosphorylation level of NF-κB and IκB-α decreased significantly, indicating that quinic acid suppressed DSS-induced colon inflammation by inhibiting the activation of the MyD88/NF-κB signaling pathway.

### 3.5. Quinic Acid Improved Gut Microbiota Composition of DSS-Induced Colitis Mice

**Figure 5:**
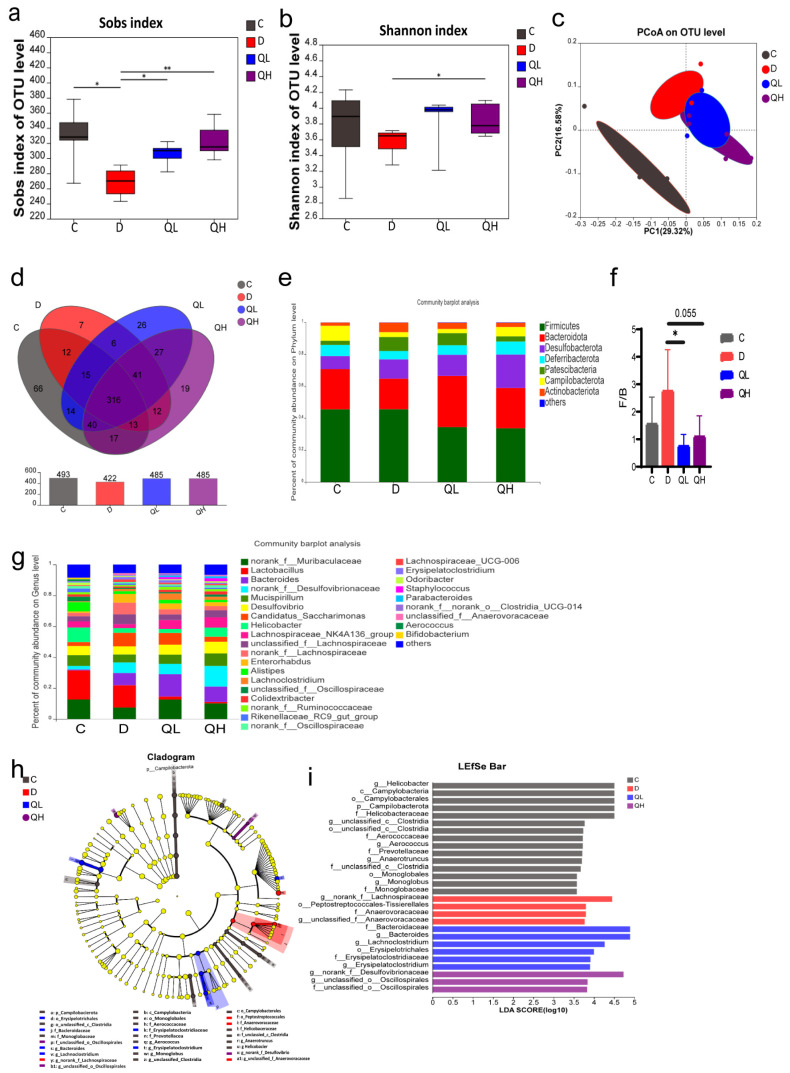
Effects of quinic acid on gut microbiota in the DSS-induced mice. (**a**) Sobs index; (**b**) Shannon index; (**c**) PCoA of gut microbiota communities based on OTU level; (**d**) Venn diagram; (**e**) column diagram of microbial composition at phylum level; (**f**) the ratio of *Firmicutes* to *Bacteroidota*; (**g**) column diagram of microbial composition at genus level; (**h**) taxonomic tree of LEfSe analysis; (**i**) histogram of LDA scores higher than 3 calculated from feature differential species. Statistical analysis, Student’s *t*-test, * *p* < 0.05, ** *p* < 0.01.

As studies have shown that the occurrence of colitis is related to the imbalance of gut microbiota [4], the regulation effect of quinic acid on gut microbiota was also analyzed in this study. The *Sobs* index reflects community richness, and the *Shannon* index reflects community diversity. Both of them indicate the α diversity of gut microbiota. As shown in Figure 5a,b, compared with group C, the α diversity of group D decreased significantly, and both the *Sobs* index and *Shannon* index increased significantly after quinic acid treatment. PCoA results based on Bray–Curtis distance showed that DSS treatment significantly changed gut microbiota, while the unweighted unifrac distance of the QL and QH groups was different from that of group D (Figure 5c), indicating that the β diversity was also changed after quinic acid treatment. The Venn diagram showed that there were 66 unique OTUs in group C, 7 unique OTUs in group D, 26 unique OTUs in group QL, and 19 unique OTUs in group QH, indicating that the types of gut microbiota were decreased by DSS induction but obviously increased after quinic acid treatment (Figure 5d).

The composition of gut microbiota in the different groups at the phylum and genus levels was further analyzed. At the phylum level, *Firmicutes*, *Bacteroidota*, *Desulfobacterota*, *Defferibacterota*, *Patescibacteria*, *Campilobacterota*, and *Actinobacteriota* were dominant (Figure 5e). The abundance of *Bacteroidota* and *Desulfobacterota* was decreased by DSS but increased by quinic acid treatment, while that of *Patescibacteria* and Actinobacteria was increased by DSS but decreased by quinic acid. The ratio of *Firmicutes* to *Bacteroidota* (F/B) was also determined, as it is considered a marker of dysbiosis of gut microbiota. The result suggested that DSS led to an increase in the F/B ratio, but quinic acid treatment significantly decreased it (Figure 5f). At the genus level, the dominant bacteria in each group varied from each other (Figure 5g).

To further investigate the changes in specific bacterial flora after quinic acid treatment, LEfSe analysis was used to detect the specific species with statistical differences in each group. As shown in Figure 5h,i, the control group was enriched in *g__Helicobacter*, *c__Campylobacteria*, *o__Campylobacterales*, etc. When treated by DSS, the specific bacteria were changed to *g__norank_f__Lachnospiraceae*, *o__Peptostreptococcales-Tissierellales*, *f__Anaerovoracaceae*, etc. However, the specific bacteria in the QL and QH groups were also different. The QL group showed significant enrichment in *f__Bacteroidaceae*, *g__Bacteroides*, etc., while the QH group was enriched in *g__norank_f__Desulfovibrionaceae*, *g__unclassified_o__Oscillospirales*, and *f__unclassified_o__Oscillospirales*. These results show that the gut microbiota of the mice with DSS-induced colitis was obviously changed after quinic acid treatment.

### 3.6. Effect of Quinic Acid on the Pathological Symptoms of Pseudo-Germ-Free Ccolitis Mice

**Figure 6:**
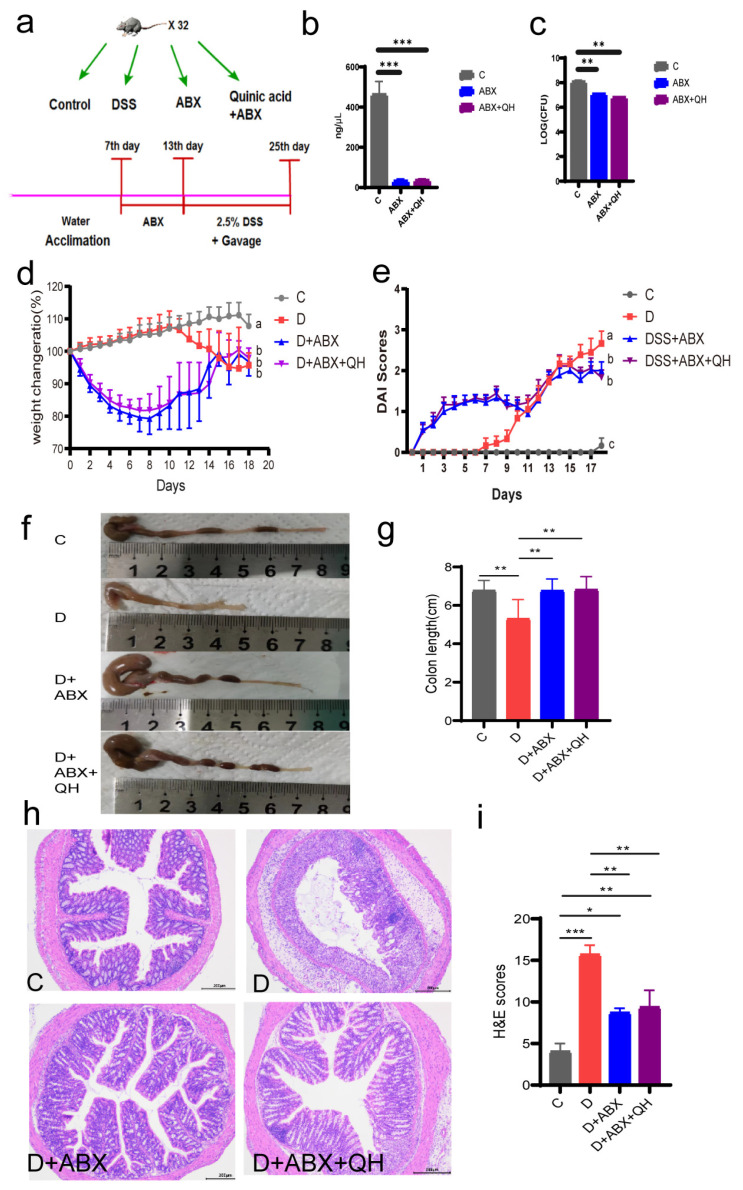
Effect of quinic acid on pathological symptoms of the pseudo-germ-free colitis mice. (**a**) Experimental scheme; (**b**) total DNA concentration in feces of ABX-treated mice; (**c**) the total number of bacterial colonies after ABX treatment; (**d**) ratio of weight change, a different letter on each line indicates significant difference at *p* < 0.05; (**e**) disease activity index (DAI), a different letter on each line indicates significant difference at *p* < 0.05; (**f**) the pictures of colons; (**g**) colon length; (**h**) H&E pictures of colons; (**i**) H&E scores. Statistical analysis, Student’s *t*-test, * *p* < 0.05, ** *p* < 0.01, *** *p* < 0.001.

To verify the roles of gut microbiota in the regulation of quinic acid on colitis, the pseudo-germ-free mice were subjected to DSS induction. The pseudo-germ-free mice model was setup by antibiotic treatment (ABX) for 7 days and then induced by DSS (Figure 6a). The total DNA concentration and colony numbers of the mouse feces after ABX for 7 days were determined. As shown in Figure 6b,c, the DNA concentration of fecal flora in the pseudo-germ-free mice (~30 ng/µL) were significantly reduced when compared with the control group (~450 ng/µL). And the number of fecal flora was also decreased compared to the C group, which was similar to previous research results [24], indicating that the pseudo-germ-free mice model was successfully setup.

The weight change in the mice is shown in Figure 6d, from which it could be observed that, compared with group C, the weight change in groups D, DSS + ABX, and DSS + ABX + QH decreased significantly, but there was no significant difference among them at the end of the induction. It is also notable that ABX treatment results in a decrease in the weight of the mice, and then, the body weight increases after the removal of antibiotics. The change trend of body weight of the DSS + ABX group and the DSS + ABX + QH group was basically the same. The DAI scores showed a similar trend (Figure 6e). Compared with group C, the DAI scores of groups D, DSS + ABX, and DSS + ABX + QH increased significantly, but there was no significant difference between the DSS + ABX and DSS + ABX + QH groups. It is also non-negligible that ABX treatment increased the DAI scores of the mice. The colon length of the group D mice was clearly shortened compared with group C, while the colon length of the DSS + ABX group and DSS + ABX + QH group was increased (Figure 6f,g). These results show that gut microbiota play important roles in the pathogenesis of colitis. When gut microbes were removed, the effects of quinic acid became unapparent.

The histological analysis of the colon tissue of the mice was analyzed by H&E staining. As shown in Figure 6h, consistent with the previous results, group C showed a normal structure, the pathological structure of the colon tissue of the mice in group C was normal, the colonic mucosal barrier was intact, and there was no ulcer and inflammatory cell infiltration. Severe acute colitis occurred in the colon of the mice treated with DSS, and the basic and crypt structures were destroyed, with more inflammatory cells infiltrated. However, in the DSS + ABX group and DSS + ABX + QH group, the pathological characteristics were improved, and the colon tissue showed an intact tissue structure with almost no obvious inflammatory infiltration. The H&E scores are shown in Figure 6i, from which the DSS significantly increased the values of H&E scores while ABX treatment significantly decreased it. However, quinic acid treatment did not decrease the value further. These results suggest that intestinal flora played essential roles during the induction of colitis by DSS, and the function of quinic acid on colitis was dependent on intestinal flora.

### 3.7. Effects of Quinic Acid on Barrier Proteins and Inflammatory Factors in Pseudo-Germ-Free Colitis Mice

**Figure 7:**
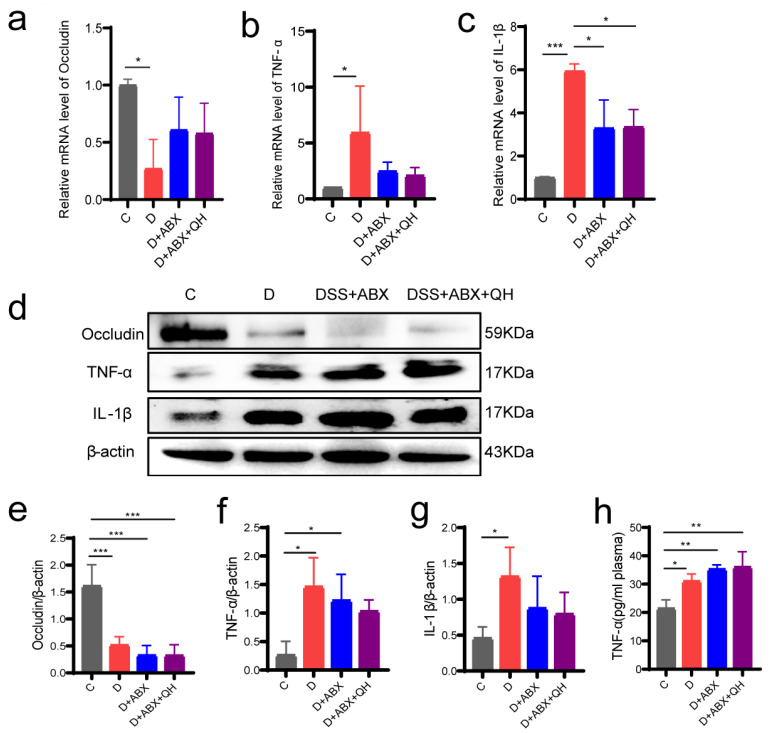
Effect of QA on the barrier function and intestinal inflammation in the pseudo-germ-free colitis mice. (**a**) Relative mRNA level of Occludin; (**b**) relative mRNA level of TNF-α; (**c**) relative mRNA level of IL-1β; (**d**) protein levels of Occludin, TNF-α, and IL-1β; (**e**) quantitative analysis of the protein level of Occludin; (**f**) quantitative analysis of the protein level of TNF-α; (**g**) quantitative analysis of the protein level of IL-1β; (**h**) protein levels of TNF-α in plasma. * *p* < 0.05, ** *p* < 0.01, *** *p* < 0.001.

The expression of barrier protein Occludin and inflammatory factors TNF-α and IL-1β at the mRNA and protein levels was analyzed by qPCR and Western blot, respectively. As shown in Figure 7a, compared with group C, the expression of Occludin was decreased in groups D, DSS + ABX, and DSS + ABX + QH. However, there was no significant difference between the DSS + ABX and DSS + ABX + QH groups. The expression of TNF-α was up-regulated in the D group and DSS + ABX group but not significantly influenced in the DSS + ABX + QH group (Figure 7b). Compared with the C group, the mRNA level of IL-1β was increased in the DSS group and decreased in the three other groups (Figure 7c). The protein level of Occludin showed similar trends with mRNA: it was down-regulated in the DSS group, DSS + ABX group, and DSS + ABX + QH group (Figure 7d,e). The detection of TNF-α and IL-1β protein levels in the colon showed that they were up-regulated after DSS treatment, and there was no significant difference among the DSS group, DSS + ABX group, and DSS + ABX + QH group (Figure 7d,f,g). The protein level of TNF-α in plasma was also detected and showed a similar result to the protein level in the colon (Figure 7h). In conclusion, these results suggest that the inhibitory effect of quinic acid on barrier function and intestinal inflammation in the colitis mice disappeared after antibiotic treatment.

## 4. Discussions

Gut microbiota are involved in the pathogenesis and development of IBD, and the modulation of gut microbiota by dietary factors shows a promising future in the treatment of IBD [25]. In this study, we found quinic acid altered the microbial structure of the feces of IBD patients by in vitro fermentation. Further determination of the roles of quinic acid by DSS-induced colitis mice models showed that quinic acid alleviated the symptoms of colitis, inhibited intestinal inflammation, and improved the dysbiosis of gut microbiota. Moreover, the role of gut microbiota during the regulation of quinic acid on colitis was confirmed by pseudo-germ-free mice.

In vitro fermentation analysis revealed that quinic acid significantly decreased the abundance of harmful bacteria, including *Escherichia-Shigella*, *Proteus*, *Paeniclostridium*, and *Streptococcus* (Figure 1f). Among them, *Escherichia-Shigella* is a Gram-negative bacterium with high abundance in IBD patients, which may be involved in destroying the tight junction of intestinal mucosal barrier and secreting lipopolysaccharides to activate Toll-like receptor 4 (TLR4), thus causing inflammation [26]. *Proteus*, a Gram-negative facultative anaerobic bacillus, is considered to be associated with CD recurrence after intestinal resection and plays a crucial role in the pathogenesis of CD by inducing inflammation [27]. *Paeniclostridium* is a spore-producing Gram-positive bacterium, which can produce toxin TcsL, causing edema, gangrene, and muscle necrosis in the human body [28]. *Streptococcus* [29] is reported to be increased in the stools of IBD patients and to be associated with tumorigenesis of IBD [30]. Quinic acid also increased the abundance of some beneficial bacteria, such as *Bifidobacterium* and *Weissella*. *Bifidobacterium* is a widely studied probiotic, and a large number of animal and clinical experiments have proved that *Bifidobacterium* can alleviate intestinal inflammation by protecting the intestinal epithelial barrier and tissue structure, reducing the production of toxic substances in patients with IBD, inhibiting the growth of potential pathogenic microorganisms, and promoting the production of short-chain fatty acids [31]. *Weissella*, belonging to the *Lactobacillaceae* family, also possesses probiotic and anti-inflammatory potential. Sandes et al. [32] found that *Weissella* could reduce intestinal permeability and depressive-like behaviors through immunomodulatory effects in mice colitis models. The increase in those genera would help reduce intestinal inflammation.

Impaired intestinal barrier and increased expression of inflammatory factors play important roles in the pathogenesis of colitis. When intestinal epithelial cells are damaged, some bacteria and antigens will enter the lamina propria of the colon, leading to the activation of the immune system [33]. Occludin is a tight junction protein forming the intestinal barrier, and TNF-α and IL-1β are the key pro-inflammatory cytokines in the pathogenesis of IBD [34]. Our results indicate that quinic acid could efficiently increase the protein level of Occludin and suppress the up-regulation of TNF-α and IL-1β induced by DSS, confirming the effects of quinic acid on intestinal barrier and inflammation. Inappropriate activation of the TLR4/MyD88/NF-κB signaling pathway is an important signaling pathway for the aggravation of intestinal inflammation in patients with IBD, and it is a common target of drug development for IBD patients [35]. The TLR4 receptor could be activated by lipopolysaccharides (LPS) of Gram-negative bacteria, and the activation of TLR4 will recruit MyD88 and result in a cascade of signal transduction, which leads to the phosphorylation and translocation of NF-κB. The translocation of pNF-κB will activate the transcription of pro-inflammatory factors TNF-α, IL-1β, etc. In this study, we found quinic acid could down-regulate the protein level of MyD88 and inhibit the phosphorylation of IKB-α and NF-κB, suggesting that quinic acid treatment could inhibit the activation of the TLR4/MyD88/NF-κB signaling pathway to suppress intestinal inflammation. The regulation of the TLR4/MyD88/NF-κB pathway by polyphenols has been reported by some studies. Huang et al. [36] found that curcumin is reported to regulate the differentiation and function of Breg cells to alleviate DSS-induced colitis by inhibiting the TLR4/MyD88/NF-κB pathway. The regulation of quinic acid on immune cells needs to be investigated further, which may provide more evidence for elaborating the regulation mechanisms of quinic acid on colitis.

The roles of gut microbiota during the regulation of colitis by quinic acid were emphatically discussed in this study. Similar to in vitro fermentation, quinic acid also increased the diversity of gut microbiota in the colitis mice and significantly improved the dysbiosis of the colitis mice (Figure 5). The ratio of *Firmicutes* to *Bacteroidota* (F/B) is considered to be a feature of dysbiosis related to diseases such as colitis and obesity [37,38]. In this study, it could be observed that DSS induction significantly increased the F/B value (Figure 5f), which is consistent with previous studies [39,40], and the ratio was decreased by quinic acid treatment, indicating the improved dysbiosis of the gut microbiota. And from the LEfSe analysis, the bacteria species are also different among those groups, further confirming the alteration of the gut microbiota. However, the specific flora species was not the same between in vitro fermentation and the DSS-induced colitis mice. It may be because the gut microbiota composition of humans and mice is different, and there are almost no common bacterial species between humans and mice. Only 4% of mouse gut microbiota genera are the same as human gut microbiota genera [41,42], which leads to different changes in gut microbiota after quinic acid treatment. In addition, the condition is more complex in the intestine than in vitro fermentation, which may also contribute to the difference between in vitro fermentation and the colitis mice.

The role of the gut microbiota in quinic acid’s regulation of colitis was further evaluated by a pseudo-germ-free colitis mice model. It is notable that ABX treatment not only resulted in a reduction in body weight but also increased the DAI scores of the mice. The gut microbiome, a dynamic microbial community residing within the gastrointestinal tract, plays a crucial role in regulating host homeostasis and physiological functions. Previous studies have confirmed that antibiotic treatment can cause weight loss by impairing gut microbiota [43,44], and our result is consistent with those studies. From the results of Figure 6, it could be observed that the DAI scores and morphological characters of the DSS + ABX mice were less severe than those of the DSS mice, suggesting that the gut microbiome might participate in the pathogenesis of colitis and the intestine is resistant to DSS induction after ABX treatment to some extent. However, the detection of the barrier protein Occludin and inflammatory factors TNF-α and IL-1β indicate the occurrence of colitis in the DSS + ABX mice. Moreover, there was no difference between the DSS + ABX group and the DSS + ABX + QH group, suggesting that the relieving effect of quinic acid on the colitis mice was inhibited after the ablation of gut microbiota. From the results of the ABX-treated mice, it is concluded that gut microbiota play important roles during the regulation of DSS-induced colitis by quinic acid. As most dietary polyphenols are metabolized by the gut microbiome into small metabolites to exert function [45], it is suspected that the metabolic transformation of quinic acid by gut microbiota is indispensable for the exertion of its function. The exact microbial metabolites of quinic acid and their regulation on the MyD88/NF-κB signaling pathway need to be investigated further.

In conclusion, we investigated the regulatory effect of quinic acid on colitis and the possible mechanisms. Our results show that quinic acid could improve the dysbiosis of gut microbiota, alleviate the pathological symptoms of DSS-induced colitis, and inhibit intestinal inflammation by suppressing the activation of the MyD88/NF-κB signaling pathway in mice colon. By ablation of gut microbiota with antibiotics, the effects of quinic acid on relieving colitis were diminished, which verified the essential role of gut microbiota. The present study confirmed the role of quinic acid in the treatment of colitis and suggested that quinic acid could be used as a potential supplement in the treatment of IBD. However, the therapeutic effects of quinic acid on IBD could be further verified by clinical trials involved in IBD patients in the future, and the microbial metabolites also need to be identified by metabolomic analysis.

## Data Availability

The original contributions presented in the study are included in the article/Supplementary Materials, further inquiries can be directed to the corresponding author.

## Supplementary Materials

The following supporting information can be downloaded at: https://www.mdpi.com/article/10.3390/foods14132267/s1, Table S1: Disease activity index scoring criteria; Table S2: Primer sequences for qPCR.
