# Supplementary material for: Millet Quinic Acid Relieves Colitis by Regulating Gut Microbiota and Inhibiting MyD88/NF-κB Signaling Pathway
Source: Foods. 2025 Jun 26;14(13):2267. doi: 10.3390/foods14132267 (PMC12249245; doi:10.3390/foods14132267)
Supplement: Supplementary file 1 [file foods-14-02267-s001.zip › foods-3569671-supplementary.pdf]

Supplementary Tables

Table S1. Disease activity index scoring criteria

Score	Weight loss (%)	Stool consistency	Occult blood or gross bleeding
0	None	Normal	Negative
1	1-5	Loose stool	Negative
2	5-10	Loose stool	Hemoccult positive
3	10-15	Diarrhea	Hemoccult positive
4	> 15	Diarrhea	Gross bleeding

Table S2. Primer sequences for qPCR

**Gene**	**Primer sequence**	
GAPDH	FP	TGGAGAAACCTGCCAAGTATGA
	RP	TGGAAGAATGGGAGTTGCTGT
TNF-α	FP	AGCCCCCAGTCTGTATCCTT
	RP	GGTCACTGTCCCAGCATCTT
IL-1β	FP	GTGCTTGTTCCTCAGCCTCT
	RP	CACCCTTCTCCAGCTGGAAG
Occludin	FP	ATCACTTTTCCTGCGGTGAC
	RP	GGAACGTGGCCGATATAATG
